# High-fat and high-cholesterol diet decreases phosphorylated inositol-requiring kinase-1 and inhibits autophagy process in rat liver

**DOI:** 10.1038/s41598-019-48973-w

**Published:** 2019-08-29

**Authors:** Hisao Naito, Yuki Yoshikawa-Bando, Yuan Yuan, Sayuki Hashimoto, Kazuya Kitamori, Hiroshi Yatsuya, Tamie Nakajima

**Affiliations:** 1 Department of Public Health, Fujita Health University School of Medicine, Toyoake, 470-1192 Japan; 2 Department of General Medicine/Family & Community Medicine, Nagoya University Graduate School of Medicine, Nagoya, 466-8550 Japan; 3 College of Life and Health Sciences, Chubu University, Kasugai, 487-8501 Japan; 4 College of Human Life and Environment, Kinjo Gakuin University, Nagoya, 463-8521 Japan

**Keywords:** Chaperone-mediated autophagy, Non-alcoholic steatohepatitis

## Abstract

Precise molecular pathways involved in the progression of non-alcoholic steatohepatitis (NASH) remain to be elucidated. As Mallory–Denk bodies were occasionally observed in the enlarged hepatocytes in NASH model rat (SHRSP5/Dmcr) fed high-fat and high-cholesterol (HFC) diet, we aimed to clarify the roles of autophagy and endoplasmic reticulum (ER) stress in NASH progression. Male SHRSP5/Dmcr were randomly divided into 4 groups. Two groups were fed a control diet; the other two groups were fed a HFC diet for 2 and 8 weeks, respectively. The HFC diet increased the autophagy-related proteins levels and microtubule-associated protein 1 light chain 3-II/I ratio after 2 and 8 weeks, respectively. However, regarding ER stress-related proteins, the HFC diet decreased the levels of phosphorylated (p-) inositol-requiring kinase-1 (p-IRE-1) and p-protein kinase RNA-like ER kinase after 2 weeks. Additionally, the HFC diet increased anti-ubiquitin-positive cells and the level of the autophagy substrate p62, suggesting that the HFC diet induced dysfunction in ubiquitin-dependent protein degradation pathways. In conclusion, the HFC diet arrested the autophagy process in the liver; this was particularly associated with decreases in p-IRE-1 expression.

## Introduction

The number of patients with non-alcoholic fatty liver disease (NAFLD) is increasing in both developed and developing countries, and this is associated with unhealthy lifestyle habits, including poor eating habits and lack of exercise^1–3^. Most NAFLD patients exhibit a non-progressive simple fatty liver without hepatitis and fibrosis. However, the condition in some patients can progress to an advanced pathological stage, and these patients are diagnosed with non-alcoholic steatohepatitis (NASH)^2^. Histopathologically, lipid droplet accumulation, inflammatory cell infiltration, hepatocyte degenerative changes, such as Mallory–Denk bodies and ballooning, and fibrosis are commonly observed in the livers of patients with NASH^2^. NASH has been shown to be related to metabolic syndrome, characterised by a constellation of metabolic risk factors such as obesity, diabetes, lipid-metabolism abnormality, and hypertension^2,3^. It can sometimes progress to conditions associated with irreversible hepatic damage, such as cirrhosis and cancer^2^; therefore, the prevention of NASH is extremely important.

One of important issues is that non-invasive differentiation between simple fatty liver and NASH is difficult with laboratory investigations of blood and several kinds of imaging approaches, such as ultrasonography, computed tomography and magnetic resonance imaging. Liver biopsy is the gold-standard method to distinguish between simple fatty liver and NASH; however, this method is invasive, and is usually difficult to be performed in standard clinical settings. Therefore, patients previously diagnosed with NAFLD are often assumed to have benign fatty liver. Thus, the development of a non-invasive and accurate diagnostic approach and the assessment of the mechanisms of NASH should be prioritised^4^. In this regard, an animal model reflecting human NASH is immediately required.

We previously developed an animal model for NASH using stroke-prone spontaneously hypertensive rats (SHRSP5/Dmcr)^5^. When these rats were fed a high-fat and high-cholesterol (HFC) diet, they showed steatohepatitis in the liver after 2 weeks, ballooning hepatocytes, Mallory–Denk bodies and fibrosis after 8 weeks and honeycomb fibrosis after 14 weeks. An HFC diet also increased the serum levels of inflammatory cytokines and tumour necrosis factor-α (TNFα) and induced a signalling network that included nuclear factor-kappa B, mitogen-activated protein kinase and nuclear factor erythroid 2-related factor 2 in the liver^6,7^. These findings suggested the enhancement of inflammatory signalling by an HFC diet. Additionally, an HFC diet increased the expressions of proteins involved in fibrosis development such as transforming growth factor-β1, alpha smooth muscle actin, and collagen type I, alpha-1^8–11^. The upregulation of these proteins was similarly observed in the serum/liver of patients NASH^1,12^. Therefore, the developed rat model is a relevant experimental model for human NASH.

Hepatocyte necrosis or apoptosis has been shown to be closely related to the pathogenesis of NASH^13,14^. TNFα promotes caspase activation, which is followed by an increase in apoptosis. In contrast, when caspase activity is depressed, the cell death mode is switched from apoptosis to necrosis, even if the TNFα level increases^15^. Indeed, in our NASH animal model (SHRSP5/Dmcr) mentioned above, an HFC diet initially increased apoptosis accompanied with caspase activity elevation at 2 weeks. However, decreased caspase activity was noted at 8 weeks, and the number of necrotic hepatocytes markedly increased, while apoptotic hepatocytes were rare and were even decreased when compared to the number at 2 weeks^16^. Thus, necrosis, but not apoptosis, is related to fibrosis progression.

Another programmed cell death mechanism mediated by autophagy has been the focus of recent studies, and this mechanism is related to signalling pathways that are different from those of apoptosis. Autophagy mainly maintains a balance between the production of cellular components and the breakdown of damaged or unnecessary organelles and other cellular constituents^17–19^. Accordingly, autophagy has attracted considerable attention as it is closely linked to many diseases including liver diseases^17^. In cytoplasm showing damage, a membrane structure termed isolation membrane appears. It is present in the initial stage of autophagosome formation, and it elongates and encloses unnecessary cytoplasmic constituents and cell organelles^19^. When an autophagosome fuses with a lysosome, the cytoplasmic constituents are degraded by lysosomal hydrolases and the degraded products are recycled. In mammalian systems, autophagy homeostatically works in fasting and well fed conditions^17^. Issues with homeostatic autophagy can result in many diseases. In liver diseases, it is well known that autophagy breakdown can induce damage^20^.

Unfolded protein response (UPR), a signal transduction system, is activated in response to endoplasmic reticulum (ER) stress, which is a condition involving the accumulation of misfolded or unfolded proteins in the ER lumen, to restore cell homeostasis^21^. However, when activated UPR is unable to maintain homeostasis, the autophagy system alternatively works to maintain homeostasis of organelles^22–24^. Thus, autophagy has attracted a lot of interest in hepatic disease progression in relation to the ER stress response.

The present study aimed to investigate the roles of autophagy and ER stress in the progression of HFC diet induced fibrotic steatohepatitis in SHRSP5/Dmcr rats.

## Results

### Levels of p62 and ubiquitin

**Figure 1:**
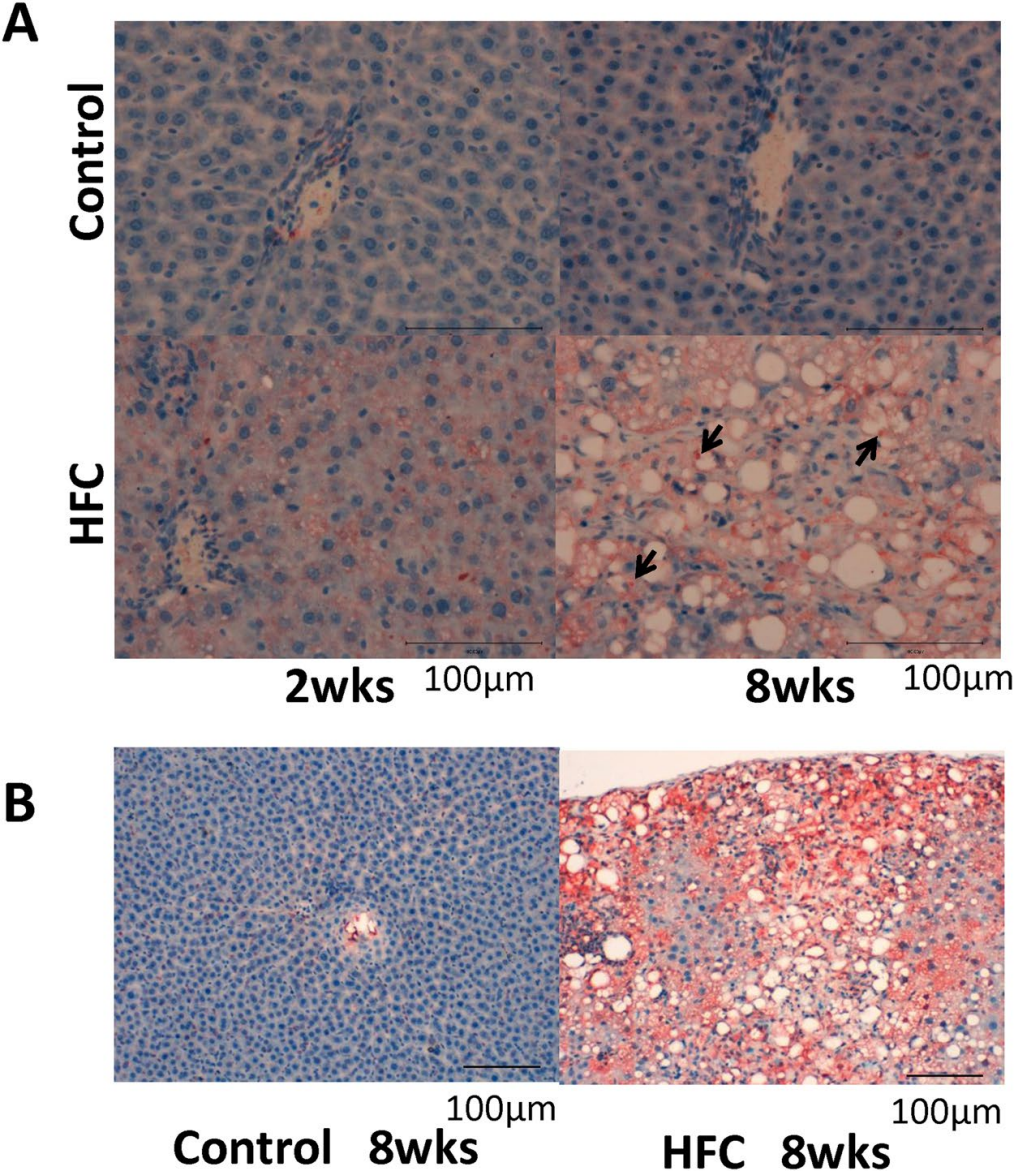
(**A**) Representative images of p62 immunostaining of samples from SHRSP5/Dmcr rats fed control and HFC diets for 2 and 8 weeks. *Arrows* indicate the accumulation in the cytoplasm of enlarged hepatocytes. *Scale bar* 100 μm. (**B**) Representative images of ubiquitin immunostaining of samples from rats fed control and HFC diets for 8 weeks. *Scale bar* 100 μm.

**Figure 2:**
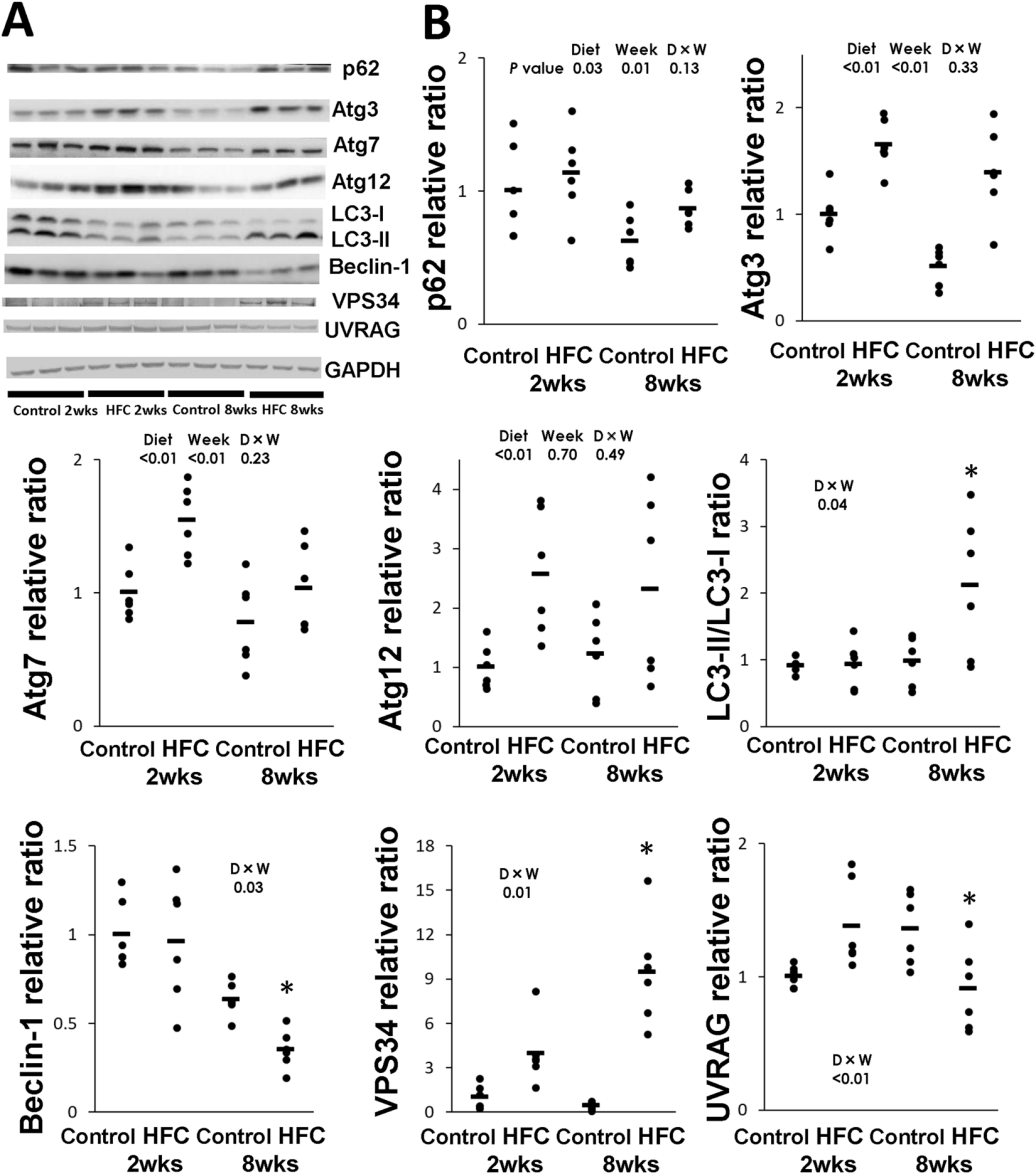
(**A**) Representative Western blot images of p62 and autophagy-related proteins (Atg) 3, 7 and 12, and microtubule-associated protein 1 light chain 3 (LC3), beclin-1, class III phosphatidylinositol 3-kinase (VPS34) and UV radiation resistance-associated gene protein (UVRAG). Samples were obtained from SHRSP5/Dmcr rats fed control and HFC diets for 2 and 8 weeks. (**B**) Data are presented as means (bar) and dot plots for individual data. Glyceraldehyde-3-phosphate dehydrogenase (GAPDH) was used as a loading control. Wide-length blots/gel image are presented in Supplementary information. Data was analysed by two-way analysis of variance between diet and feeding duration (week). *P* values of diet, and week, D × W (diet × week interaction effect) were expressed within each graph. *Significant difference between HFC diet and control-diet groups, *P* < 0.05.

p62 (sequestosome1/SQSTM1/A170) has been reported to bind polyubiquitin noncovalently and its overexpression results in large cytoplasmic aggregates^25^. p62 directly binds to microtubule-associated protein 1 light chain 3 (LC3) localised in autophagosome membranes. No p62-positive areas were observed in the livers of rats from the control-diet groups. However, a p62-positive area was clearly observed in the livers of rats from the 2-week HFC-diet group, and the area was more prominent in the livers of rats from the 8-week HFC-diet group, especially in the cytoplasm of enlarged hepatocytes (Fig. 1A). Western blot analysis showed that the p62 level was significantly higher in the livers of rats from the HFC-diet group than in the livers of rats from the control-diet group (Fig. 2); however, no significant interaction was observed between effect of diet and that of feeding duration. As p62 is a substrate of autophagy, the increase in its level may indicate the inhibition of autophagy induced by the HFC diet.

Ubiquitin is a small (8.5 kDa) regulatory protein that is found in almost all tissues (ubiquitously) of eukaryotic organisms^26^. The ubiquitin pathway has been implicated in the pathogenesis of many human diseases. The HFC diet significantly increased the ubiquitin-positive area in the livers of rats, suggesting the accumulation of abnormal proteins (Fig. 1B).

### Autophagy-related molecules

Several kinds of autophagy-related (Atg) proteins are required for autophagosome formation^27^. The LC3 system is also important for transport and maturation of autophagosomes^18,27^. Once an autophagosome has matured, its external membrane fuses with lysosomes and its components are degraded^28^. In the present study, the levels of Atg3, Atg7 and Atg12 were significantly higher in the livers of rats from the HFC-diet group than in the livers of rats from control-diet group (Fig. 2); however, no significant interactions were observed between diet and feeding duration effect. On the contrary, the LC3-II/I ratio was significantly higher in the livers of rats from the 8-week HFC-diet group than in the livers of rats from the 8-week control-diet group. However, there was no difference in the level between the 2-week HFC-diet group and 2-week control-diet group.

The beclin-1 protein participates in autophagosome formation, and conflicting theories of the enhancement or suppression of autophagosome–lysosome fusion by beclin-1 have been reported^29^. Interaction between the HFC diet and feeding duration effect was significantly observed in the level of beclin-1, and the level was lower in the livers of rats from the 8-week HFC-diet group than in those of rats from the 8-week control-diet group. However, there was no difference in the level between the 2-week HFC-diet group and 2-week control-diet group. Similarly, the interaction between diet and feeding duration effect was significantly observed in the level of class III phosphatidylinositol 3-kinase (VPS34), which forms a complex with beclin-1, whereas the level was higher in the livers of rats from the 2-week and 8-week HFC-diet groups than in the livers of rats from the corresponding control-diet groups. However, significant interaction between diet and feeding duration effect was observed in the level of UV radiation resistance-associated gene protein (UVRAG), which is part of the beclin-1 complex, and the level was lower in the livers of rats from the 8-week HFC-diet group than in the livers of rats from the 8-week control diet group; however, there were no differences in expression between the 2-week HFC-diet group and the 2-week control-diet group.

### Electron microscopy

**Figure 3:**
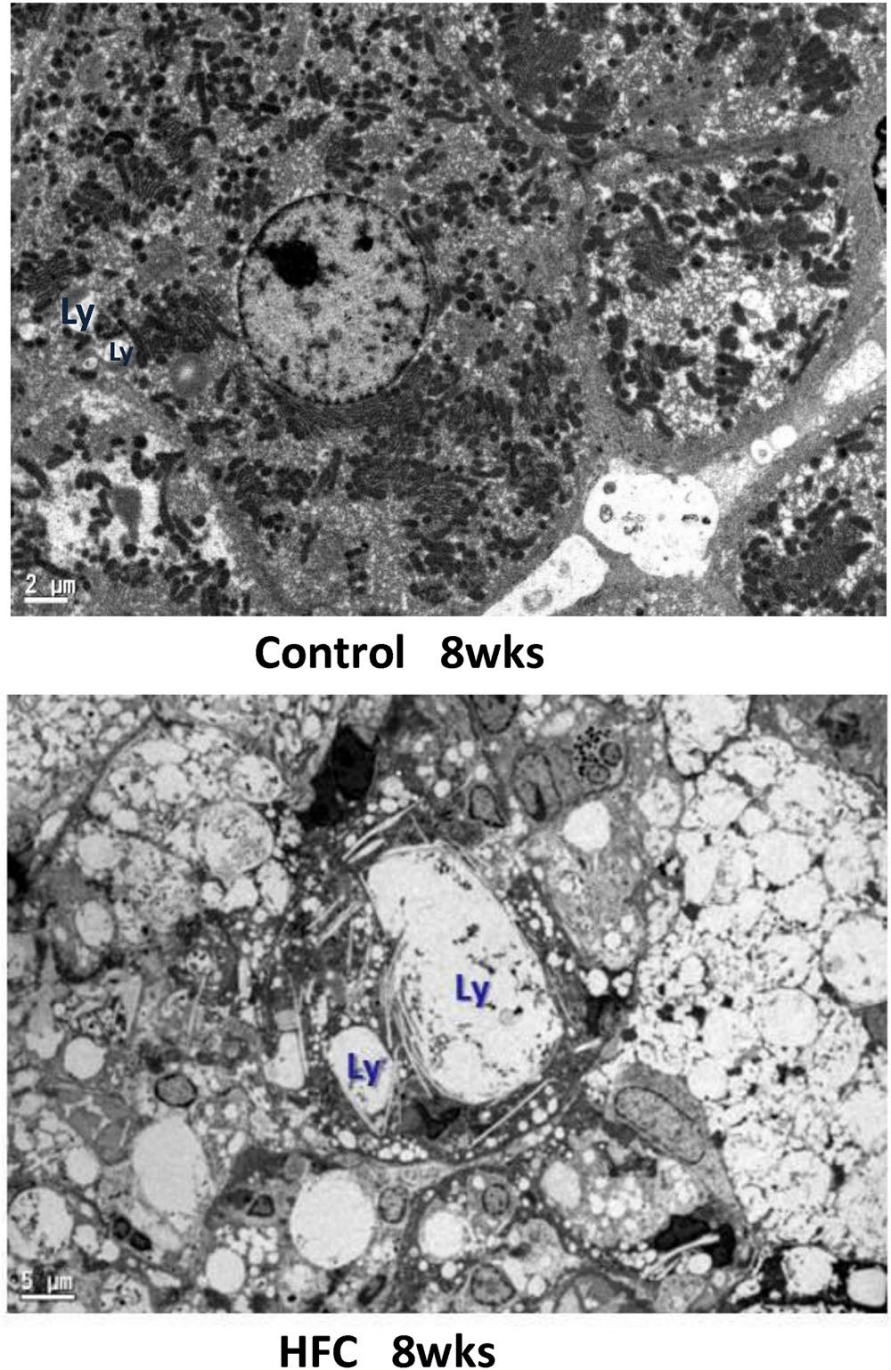
Electron microscopic image. Giant lysosomes (*arrow*) and accumulation of autophagosomes (top-right), related to autologous cells with heterochromatin that adhered to the nuclear membrane, are seen in the livers of rats fed the HFC diet for 8 weeks. *Scale bar* 2 μm (control-diet group) and 5 μm (HFC-diet group).

Electron microscopy showed giant lysosomes which are not normally recognized, and accumulation of autophagosomes (not typical), which were related to autologous cells with heterochromatin that adhered to the nuclear membrane, in the livers of rats from the 8-week HFC-diet group (Fig. 3). On the contrary, normal lysosomes with vacuolar degeneration are recognised in the liver of rats from the 8-week control-diet group.

### ER stress-related signal transduction

**Figure 4:**
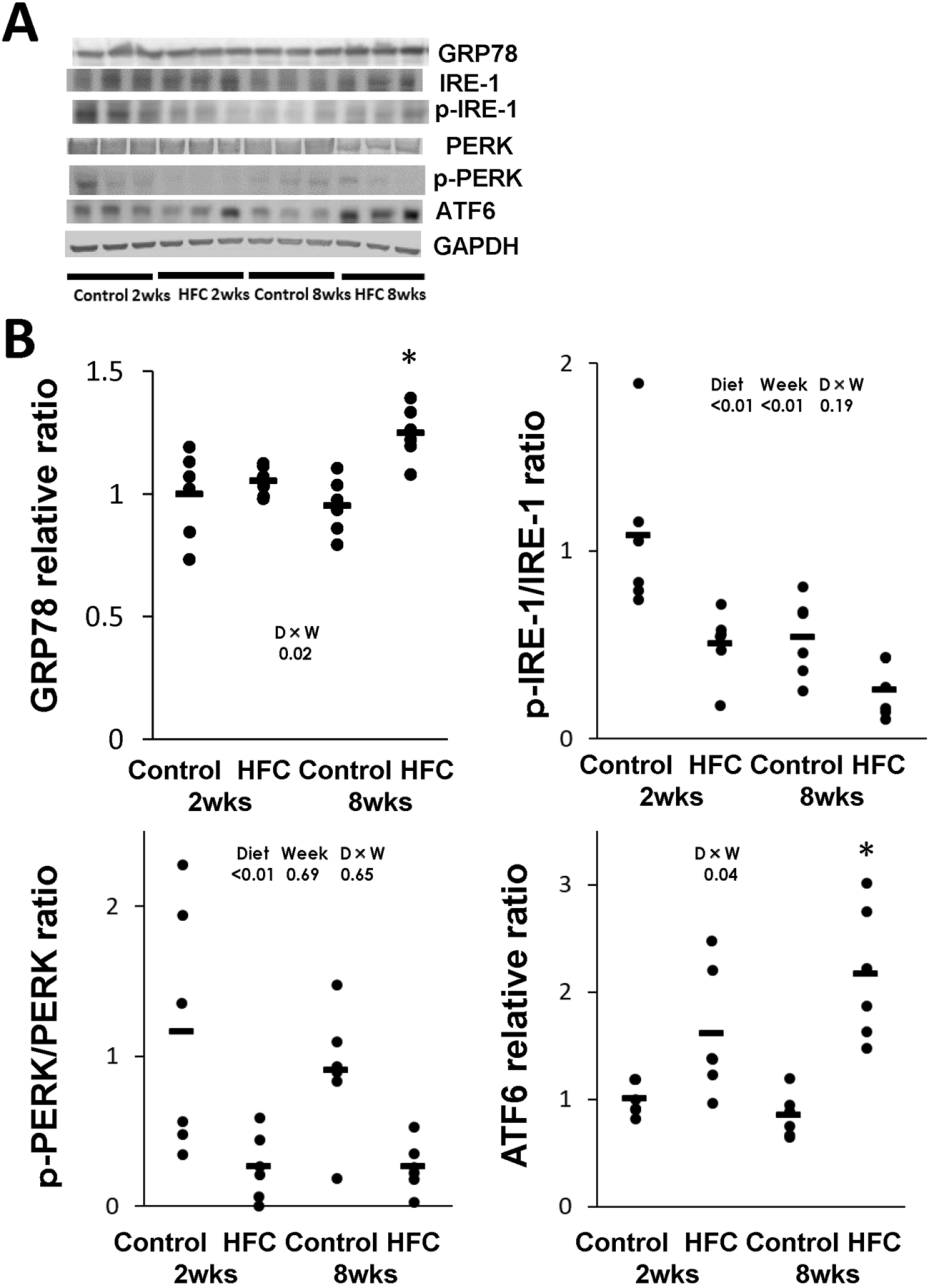
(**A**) Representative Western blot images of the endoplasmic reticulum (ER) stress-related proteins binding immunoglobulin protein/glucose-regulated protein-78 (GRP78), phosphorylated (p-) inositol-requiring enzyme 1 (p-IRE-1)/IRE-1 ratio, p-protein kinase RNA-like ER kinase (p-PERK)/PERK ratio and activating transcription factor-6 (ATF6). (**B**) Data are presented as means (bar) and dot plots of individual data. GAPDH was used as a loading control. Wide-length blots/gel image are presented in Supplementary information. Data was analysed by two-way analysis of variance between diet and feeding duration (week). *P* values of diet, week, D × W (diet × week interaction effect) were expressed within in each graph. *Significant difference between the HFC diet and control-diet groups, *P* < 0.05.

Accumulation of unfolded proteins in the ER activates the stress sensors IRE-1, protein kinase RNA-like ER kinase (PERK) and activating transcription factor-6 (ATF6) localised on the membrane^23^. We measured the protein expressions of phosphorylated (p-) IRE-1/IRE-1 ratio, p-PERK/PERK ratio, ATF6, and binding immunoglobulin protein/glucose-regulated protein-78 (GRP78), which are associated with the accumulation of unfolded proteins in the ER, in the livers of rats fed the control and HFC diets. Significant interaction between diet and feeding duration effect was observed in the expressions of GRP78 and ATF6, and the expressions were higher in the livers of rats from the 8-week HFC-diet group than in the livers of rats from the 8-week control-diet group; however, there was no difference in the expression between the 2-week HFC-diet group and 2-week control-diet group (Fig. 4). On the contrary, the expressions of p-PERK/PERK and p-IRE-1/PERK were significantly lower in the livers of rats from the HFC-diet groups than in the livers of rats from the control-diet groups; however, no significant interaction was observed between diet and feeding duration effect.

## Discussion

The present study showed that an HFC diet increased the expressions of autophagy-related signalling such as Atg factors, LC3-II/I and VPS34, which appeared to enhance autophagy signalling, while it suppressed the expression of p-IRE-1, p-PERK and beclin-1, suggesting that the diet can inhibit autophagosome–lysosome fusion and proteasomal degradation. Thus, an HFC diet might result in abnormal giant lysosome formation, and p62 protein and Mallory–Denk body accumulation.

LC3-II is used as a marker of autophagy^30^. In the present study, an HFC diet increased the expressions of Atg proteins in the livers of rats at 2 and 8 weeks and LC3-II/I at 8 weeks, suggesting the enhancement of signalling for autophagosome formation in the liver.

Beclin-1 forms a complex with VPS34 and UVRAG and enhances phagosome formation^29^. Conversely, when beclin-1 forms a complex with B-cell lymphoma 2 (Bcl-2) family proteins such as Bcl-2 and Bcl-xL, the formation of the beclin-1-VPS34 complex is inhibited, and thus, autophagy is inhibited^31,32^. Yetti *et al*. reported that an HFC diet suppressed the expression of Bcl-2 and Bcl-xL in livers of SHRSP5/Dmcr rats^16^, suggesting induced formation of the beclin-1-VPS34 complex. In the present study, the HFC diet increased the expression of VPS34, but suppressed the expressions of beclin-1 and UVRAG at 8 weeks, which participates in autophagosome–lysosome fusion^33^. Therefore, the effect of this signalling pathway autophagosome formation with HFC diet is established, but the effect of the autophagosome–lysosome fusion might not be established.

The ER participates in the synthesis, folding and transport of proteins, and any dysfunction can lead to the retention of inactive proteins in the ER lumen, which is referred to as ER stress^34^. Signalling pathways related to the UPR are activated by ER stress. These pathways have been implicated in the impediment of protein translation and production of chaperons to attenuate ER stress^21,35^. When the UPR is unable to completely respond to ER stress, the autophagy system, which can address ER stress, is activated^22^. Accumulation of abnormal proteins in ER is believed to induce the molecule chaperone GRP78^34,36,37^. In this study, the HFC diet significantly increased the GRP78 level at 8 weeks, suggesting that ER stress was caused by the HFC diet. The three transmembrane proteins PERK, ATF6 and IRE-1 are involved in UPR function^36^. When ER stress accumulates, the PERK pathway regulates protein translation to suppress further accumulation of unfolded proteins in the ER^38^. Additionally, ATF6 senses the accumulation of abnormal proteins in the ER^39^. In our study, the HFC diet suppressed the p-PERK level in the liver, suggesting that protein translation was not prevented, and thus, the accumulation of unfolded proteins was enhanced. However, the HFC diet increased the expression of ATF6 in the livers of rats. Thus, the HFC diet might induce the refolding of proteins and attenuation of ER stress by increasing the production of chaperone proteins. Similar results had also been reported in NASH studies^40,41^.

Accumulations of unfolded proteins that are not completely removed by UPR are removed via the IRE-1 pathway. If this process is also unable to completely remove unfolded proteins, UPR induces apoptosis and autophagic processes^38,39,42^ The IRE-1 pathway is also downstream of LC3, which activates autophagy^37^. In IRE-1 knockout mouse cells, although LC3 processing occurs, normal autophagosomes are not formed, therefore autophagy could not occur, whereas in PERK-deficient cells and ATF6 knockdown cells, autophagy was induced after ER stress in a manner similar to that of the wild-type cells^37^. Therefore, autophagosomes cannot form without the IRE-1 pathway (i.e., presence of p-IRE-1). Additionally, in the present study, although the HFC diet increased the LC3-II/I ratio at 8 weeks, p-IRE-1 was suppressed from 2 weeks; therefore, abnormal giant lysosomes were observed near the accumulation of autophagosomes, suggesting that the HFC diet suppressed autophagosome–lysosome fusion. In fact, the accumulation of autophagosomes identified on electron microscopy is a hallmark of blockade of the autophagic flux^40,43^.

The HFC diet promoted the activation of caspases 3/7, 8 and 9 at 2 weeks and slightly increased apoptotic hepatocytes, but it decreased the activities of these caspases at 8 weeks of feeding in SHRSP5/Dmcr rats^16^. Thus, the number of necrotic hepatocytes dramatically increased at 8 weeks of HFC feeding and fibrosis occurred at the same time^16^. In the present study using this model rat, the HFC diet increased the level of p62, which is decomposed by the autophagy pathway. Additionally, immunohistological staining using anti-ubiquitin antibody showed the accumulation of ubiquitin in the livers of rats fed the HFC diet for 8 weeks. p62 is a polyubiquitin chain-binding protein involved in ubiquitin-proteasome pathway^26,44,45^. This protein selectively recognises autophagic components and mediates the movement of such components into autophagosomes by directly binding to a small ubiquitin-like modifier^25,46^. When autophagy is selectively suppressed, excessive p62 protein accumulates, resulting in the formation of an ubiquitin-p62 inclusion body^45,47^. Taken together, the findings suggested that the HFC diet suppressed the autophagy process in the livers of rats. As mentioned above, the HFC diet slightly induced apoptosis at 2 weeks, whereas the HFC diet suppressed at 8 weeks^16^. On the contrary, the HFC diet promoted necrosis at 2 weeks and further increased the number of necrotic cells at 8 weeks. Therefore, this diet initially suppressed autophagy, then suppressed apoptotic cell death and finally dramatically induced necrotic cell death. All these processes resulted in hepatocyte degeneration and fibrosis development.

It is important to discuss the relationship between autophagy and Mallory–Denk bodies, which are hepatocyte cytoplasmic inclusions found in several liver diseases and consist primarily of the ubiquitin-binding protein p62 and the cytoskeletal proteins 8 and 18 (CK8/18)^48,49^. These bodies accumulated in the livers of rats fed the HFC diet for 8 weeks. Generally, it is difficult to degrade large aggregates of denatured proteins with proteasomes, but they can be eliminated by autophagy^44^. Thus, the p62 and CK8/18 proteins in Mallory–Denk bodies can be decomposed by autophagy. Therefore, the suppression of autophagy contributed to Mallory–Denk body formation^44^. Mallory–Denk bodies are found in not only NASH but also in several other liver diseases, such as chronic alcoholic steatohepatitis^50,51^. Previous studies found that alcohol induced the production of CK8/18 and p62, and thereby promoted the formation of Mallory–Denk bodies^52^.This finding is similar to the finding of the present study, as the HFC diet significantly induced increases in hepatic p62 and serum CK18 and a decrease in hepatic CK18^16^, and induced Mallory–Denk bodies.

In conclusion, an HFC diet induced ER stress because of accumulation of unfolded proteins in the livers of SHRSP5/Dmcr rats through the inhibition of IRE-1 and PERK pathway activation. Moreover, this diet suppressed the expression of p-IRE-1, leading to the failure of autophagosome–lysosome fusion. Furthermore, this diet disturbed the autophagy process at an early stage and promoted the development of steatohepatitis and fibrosis in SHRSP5/Dmcr rats.

## Methods

### Animal diets

Control diet (stroke-prone control chow diet, 20.8% protein, 4.8% fat, 3.2% fibre and 58.2% carbohydrate) in Supplementary information, and HFC diet (68% control diet, 25% palm oil, 5% cholesterol and 2% colic acid)^5^ were purchased from Funabashi Farm (Chiba, Japan).

### Animal experiments

All animal experiments were performed in compliance with the guidelines for animal experiments proposed by Kinjo Gakuin University Animal Center. The study protocol was approved by the Committee on Ethics of Animal Experiments of Kinjo Gakuin University Animal Center (approval nos. 27 and 65). Male offspring of SHRSP5/Dmcr rats were obtained by mating male and female SHRSP5/Dmcr rats with high serum total cholesterol levels as previously described^5^. All rats were housed in a temperature- and light-controlled environment (temperature, 23 ± 2 °C; humidity, 55% ± 5%; light/dark cycle, 12 h) with access to the control diet and tap water ad libitum.

Male rats were randomly divided into four groups (*n* = 6 in each group) at 10 weeks of age. Two groups were fed the control diet for 2 and 8 weeks, respectively, while the other two groups were fed the HFC diet for 2 and 8 weeks, respectively. All rats had access to the respective test diet and water ad libitum. After 18–20 h of fasting from the last feeding, the rats were weighed, anesthetised using pentobarbital (70 mg/kg) and sacrificed. the livers were removed. A portion of each liver was fixed in 10% buffered formalin for histopathological analysis, and fresh tissue was stored at −80 °C until use.

### Histopathological investigation

Electron microscopic investigations were conducted after fixing the two samples each at 8-week control and HFC groups in Karnovsky’s fixative and assessments were performed by a specialist (Kyodo Byori, Inc., Kobe, Japan).

Immunostaining was performed using 4% paraformaldehyde-fixed, paraffin-embedded tissue sections. Endogenous peroxidase was inactivated with 3% hydrogen peroxide in methanol for 15 min, according to the manufacturer’s instructions (Nichirei Biosciences Inc., Tokyo, Japan). Heat-induced antigen retrieval was performed, and it involved the use of a pressure cooker (Pearl Metal Co. Ltd., Sanjo, Japan) for 5 min and natural cooling for 40 min. The soaking solution was 10-mM citrate buffer (pH 6.0). After washing the sections with phosphate-buffered saline (PBS), they were incubated overnight at 4 °C with anti-SQSTM1/A170/p62 (018–22141, Wako, Osaka, Japan) or anti-ubiquitin (#3933, Cell Signaling Technology, Beverly, MA) antibodies. The sections were then incubated at room temperature with the secondary antibody Simple Stain Rat MAX PO (MULTI) (Nichirei Biosciences Inc). After washing the sections with PBS, colour was developed using Simple Stain AEC Solution (Nichirei Biosciences Inc). The stained sections were examined under light microscope using the DMD108 and DM750 systems (Leica, Wetzlar, Germany).

### Western blot analysis

Aliquots of liver tissue were homogenised using 3 volumes of 0.25 M sucrose–10 mM PBS (pH 7.4). The homogenates were sonicated and centrifuged at 700 × g for 10 min, and the supernatants were used as samples for Western blot analysis. The prepared samples were subjected to 8%, 10% or 14% sodium dodecyl sulphate-polyacrylamide gel electrophoresis, as previously described^53^. The transferred membrane from the gel was cut by referring to molecular weight provided by each antibody company. After blocking the membranes with 5% skim milk, each membrane was incubated with the following antibodies: SQSTM1/A170/p62 (018–22141, Wako); ATF6 (ab11909) and GRP78 (ab21685), p-IRE-1 (ab48187, Abcam plc, Cambridge, UK); Atg3 (#3415), Atg7 (#2631), Atg12 (#4180), beclin-1 (#3495), LC3-I (#4599), LC3-II (#3868), PERK (#3192), p-PERK (#3179) and VPS34, (#4263, Cell Signaling Technology); IRE-1 (pab13424, Abnova, Taipei, Taiwan) and UVRAG (19571-1-AP, Proteintech Japan, Tokyo, Japan). As loading controls for liver homogenates, glyceraldehyde-3-phosphate dehydrogenase (GAPDH) (M171-7, MBL, Nagoya, Japan) was used. Each membrane was then incubated with a secondary antibody conjugated to horseradish peroxidase for 1 h at room temperature. Specific proteins were detected using 1-Step Ultra TMB-Blotting Solution (Thermo Fisher Scientific, Inc., Waltham, MA) or ECL Western Blotting Detection Reagent (GE Healthcare, Buckinghamshire, UK), and each band was read using ImageQuant LAS 4010 (GE Healthcare). The band was analysed by densitometry, using the CS analyzer Ver. 3.0 (ATTO Corp, Tokyo, Japan). We calculated the ratio of band strength of HFC diet for 2 and 8 weeks and that of the control diet for the 8-week group to the average strength (as a 1.0) of control diet for 2 weeks on the same sheet.

### Statistical analysis

Data analysis was performed using Stata 15 statistical software package (Stata Crop, College Station, TX). The results of Western blot analysis are presented as mean (bar) and dot plots. All data were analysed using two-way analysis of variance between diet and feeding duration. When the interaction was statistically significant, the Bonferroni method as a post-hoc test was performed to compare rats that received the HFC diet and those that received the control diet. A *P* value of <0.05 was considered statistically significant. Statistical analysis of non-normally distributed data was performed after log transformation of each value.

## Supplementary information

Supplementary Information Control diet ingredients and wide-length blots/gel images. 

## References

[1] Angulo P (2002). Nonalcoholic fatty liver disease. N Engl J Med.

[2] Farrell GC, Larter CZ (2006). Nonalcoholic fatty liver disease: from steatosis to cirrhosis. Hepatology.

[3] Lazo M (2013). Prevalence of nonalcoholic fatty liver disease in the United States: the Third National Health and Nutrition Examination Survey, 1988–1994. Am J Epidemiol.

[4] Wieckowska A, Feldstein AE (2008). Diagnosis of nonalcoholic fatty liver disease: invasive versus noninvasive. Semin Liver Dis.

[5] Kitamori K (2012). Development of novel rat model for high-fat and high-cholesterol diet-induced steatohepatitis and severe fibrosis progression in SHRSP5/Dmcr. Environ Health Prev Med.

[6] Jia X (2012). The modulation of hepatic adenosine triphosphate and inflammation by eicosapentaenoic acid during severe fibrotic progression in the SHRSP5/Dmcr rat model. Life Sci.

[7] Yuan Yuan, Naito Hisao, Jia Xiaofang, Kitamori Kazuya, Nakajima Tamie (2017). Combination of Hypertension Along with a High Fat and Cholesterol Diet Induces Severe Hepatic Inflammation in Rats via a Signaling Network Comprising NF-κB, MAPK, and Nrf2 Pathways. Nutrients.

[8] Moriya T (2012). Simultaneous changes in high-fat and high-cholesterol diet-induced steatohepatitis and severe fibrosis and those underlying molecular mechanisms in novel SHRSP5/Dmcr rat. Environ Health Prev Med.

[9] Yetti H (2018). Bile acid detoxifying enzymes limit susceptibility to liver fibrosis in female SHRSP5/Dmcr rats fed with a high-fat-cholesterol diet. PLoS One.

[10] Tamada H (2016). Efficacy of Dietary Lipid Control in Healing High-Fat and High-Cholesterol Diet-Induced Fibrotic Steatohepatitis in Rats. PLoS One.

[11] Naito H (2016). Importance of detoxifying enzymes in differentiating fibrotic development between SHRSP5/Dmcr and SHRSP rats. Environ Health Prev Med.

[12] Washington K (2000). Hepatic stellate cell activation in nonalcoholic steatohepatitis and fatty liver. Hum Pathol.

[13] Guicciardi ME, Malhi H, Mott JL, Gores GJ (2013). Apoptosis and necrosis in the liver. Compr Physiol.

[14] Ribeiro PS (2004). Hepatocyte apoptosis, expression of death receptors, and activation of NF-kappaB in the liver of nonalcoholic and alcoholic steatohepatitis patients. Am J Gastroenterol.

[15] Morioka S (2014). TAK1 kinase switches cell fate from apoptosis to necrosis following TNF stimulation. J Cell Biol.

[16] Yetti H (2013). High-fat-cholesterol diet mainly induced necrosis in fibrotic steatohepatitis rat by suppressing caspase activity. Life Sci.

[17] Mizushima N, Komatsu M (2011). Autophagy: renovation of cells and tissues. Cell.

[18] Mizushima N, Yoshimori T, Ohsumi Y (2011). The role of Atg proteins in autophagosome formation. Annu Rev Cell Dev Biol.

[19] Tooze SA, Yoshimori T (2010). The origin of the autophagosomal membrane. Nat Cell Biol.

[20] Jiang P, Mizushima N (2014). Autophagy and human diseases. Cell Res.

[21] Marciniak SJ, Ron D (2006). Endoplasmic reticulum stress signaling in disease. Physiol Rev.

[22] Sakaki K, Kaufman RJ (2008). Regulation of ER stress-induced macroautophagy by protein kinase C. Autophagy.

[23] Szegezdi E, Logue SE, Gorman AM, Samali A (2006). Mediators of endoplasmic reticulum stress-induced apoptosis. EMBO Rep.

[24] Zhang J, Morris MW, Dorsett-Martin WA, Drake LC, Anderson CD (2013). Autophagy is involved in endoplasmic reticulum stress-induced cell death of rat hepatocytes. J Surg Res.

[25] Johansen T, Lamark T (2011). Selective autophagy mediated by autophagic adapter proteins. Autophagy.

[26] Reinstein E, Ciechanover A (2006). Narrative review: protein degradation and human diseases: the ubiquitin connection. Ann Intern Med.

[27] Sou YS (2008). The Atg8 conjugation system is indispensable for proper development of autophagic isolation membranes in mice. Mol Biol Cell.

[28] Seglen PO, Bohley P (1992). Autophagy and other vacuolar protein degradation mechanisms. Experientia.

[29] Itakura E, Kishi C, Inoue K, Mizushima N (2008). Beclin 1 forms two distinct phosphatidylinositol 3-kinase complexes with mammalian Atg14 and UVRAG. Mol Biol Cell.

[30] Kabeya Y (2000). LC3, a mammalian homologue of yeast Apg8p, is localized in autophagosome membranes after processing. EMBO J.

[31] Maejima Y (2013). Mst1 inhibits autophagy by promoting the interaction between Beclin1 and Bcl-2. Nat Med.

[32] Pattingre S (2005). Bcl-2 antiapoptotic proteins inhibit Beclin 1-dependent autophagy. Cell.

[33] Liang C (2008). Beclin1-binding UVRAG targets the class C Vps complex to coordinate autophagosome maturation and endocytic trafficking. Nat Cell Biol.

[34] Oslowski CM, Urano F, Measuring ER (2011). stress and the unfolded protein response using mammalian tissue culture system. Methods Enzymol.

[35] Ron D, Walter P (2007). Signal integration in the endoplasmic reticulum unfolded protein response. Nat Rev Mol Cell Biol.

[36] Kaufman RJ (1999). Stress signaling from the lumen of the endoplasmic reticulum: coordination of gene transcriptional and translational controls. Genes Dev.

[37] Ogata M (2006). Autophagy is activated for cell survival after endoplasmic reticulum stress. Mol Cell Biol.

[38] Shi Y (1998). Identification and characterization of pancreatic eukaryotic initiation factor 2 alpha-subunit kinase, PEK, involved in translational control. Mol Cell Biol.

[39] Sidrauski C, Chapman R, Walter P (1998). The unfolded protein response: an intracellular signalling pathway with many surprising features. Trends Cell Biol.

[40] Gonzalez-Rodriguez A (2014). Impaired autophagic flux is associated with increased endoplasmic reticulum stress during the development of NAFLD. Cell Death Dis.

[41] Lee S, Kim S, Hwang S, Cherrington NJ, Ryu DY (2017). Dysregulated expression of proteins associated with ER stress, autophagy and apoptosis in tissues from nonalcoholic fatty liver disease. Oncotarget.

[42] Yoshida H, Matsui T, Yamamoto A, Okada T, Mori K (2001). XBP1 mRNA is induced by ATF6 and spliced by IRE1 in response to ER stress to produce a highly active transcription factor. Cell.

[43] Li Y (2017). Autophagy mediated by endoplasmic reticulum stress enhances the caffeine-induced apoptosis of hepatic stellate cells. Int J Mol Med.

[44] Harada M, Hanada S, Toivola DM, Ghori N, Omary MB (2008). Autophagy activation by rapamycin eliminates mouse Mallory-Denk bodies and blocks their proteasome inhibitor-mediated formation. Hepatology.

[45] Korolchuk VI, Mansilla A, Menzies FM, Rubinsztein DC (2009). Autophagy inhibition compromises degradation of ubiquitin-proteasome pathway substrates. Mol Cell.

[46] Weidberg H, Shvets E, Elazar Z (2011). Biogenesis and cargo selectivity of autophagosomes. Annu Rev Biochem.

[47] Komatsu M (2007). Homeostatic levels of p62 control cytoplasmic inclusion body formation in autophagy-deficient mice. Cell.

[48] Hanada S (2012). Aging modulates susceptibility to mouse liver Mallory-Denk body formation. J Histochem Cytochem.

[49] Strnad P (2007). Transglutaminase 2 regulates mallory body inclusion formation and injury-associated liver enlargement. Gastroenterology.

[50] Sanyal AJ (2011). Endpoints and clinical trial design for nonalcoholic steatohepatitis. Hepatology.

[51] Strnad P (2013). Broad spectrum of hepatocyte inclusions in humans, animals, and experimental models. Compr Physiol.

[52] Rautou PE (2010). Autophagy in liver diseases. J Hepatol.

[53] Jia X (2013). Dysregulated bile acid synthesis, metabolism and excretion in a high fat-cholesterol diet-induced fibrotic steatohepatitis in rats. Dig Dis Sci.

