# Supplementary material for: High-fat and high-cholesterol diet decreases phosphorylated inositol-requiring kinase-1 and inhibits autophagy process in rat liver
Source: Sci Rep. 2019 Aug 29;9:12514. doi: 10.1038/s41598-019-48973-w (PMC6715744; doi:10.1038/s41598-019-48973-w)
Supplement: Supplementary file 1 — Supplementary Information Control diet ingredients and wide-length blots/gel images. [file 41598_2019_48973_MOESM1_ESM.pdf]

Title: High-fat and high-cholesterol diet decreases phosphorylated inositol-requiring kinase-1 and inhibits autophagy process in rat liver

Author List: Hisao Naito, Yuki Yoshikawa-Bando, Yuan Yuan, Sayuki Hashimoto, Kazuya Kitamori, Hiroshi Yatsuya, Tamie Nakajima

Supplementary information table 1. Control diet ingredients

Per 100 g of control diet	
Crude protein	20.8 g
Crude lipid	4.8 g
Crude fiber	3.2 g
Crude ash	5.0 g
Moisture	8.0 g
Carbohydrate	58.2 g
Vitamin A	1000 IU
Vitamin D3	200 IU
Vitamin E	5.0 mg
Vitamin K3	0.5 mg
Vitamin B1	1.0 mg
Vitamin B2	1.1 mg
Vitamin B12	1.7 µg
Niacin	10.0 mg
Calcium pantothenate	1.6 mg
Folic acid	0.1 mg
Biotin	0.03 µg
Choline	0.23 g
Inositol	900 mg

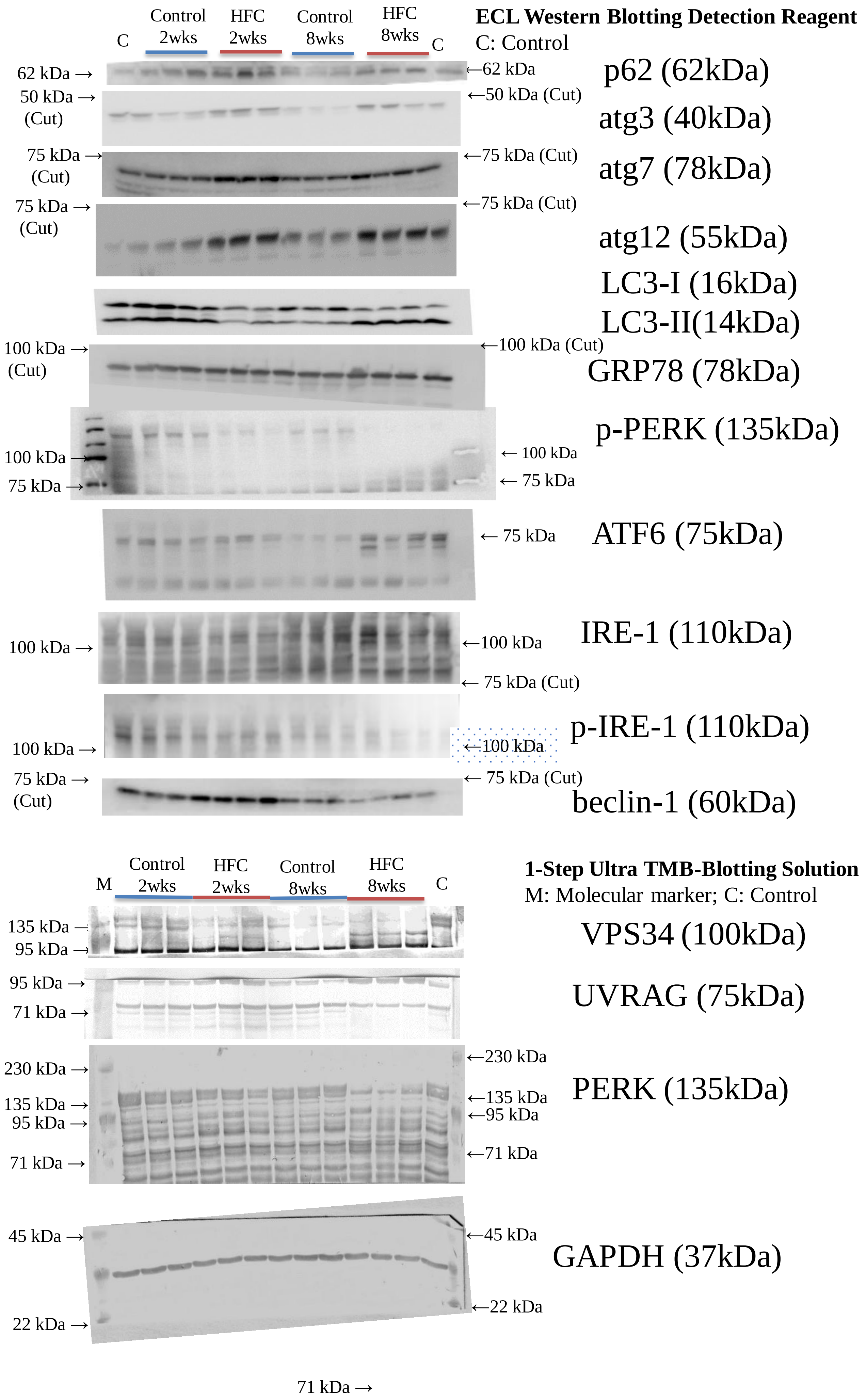
ECL Western Blotting Detection Reagent
C: Control
C
Control 2wks
HFC 2wks
Control 8wks
HFC 8wks
C
62 kDa →
←62 kDa
p62 (62kDa)
50 kDa → (Cut)
←50 kDa (Cut)
atg3 (40kDa)
75 kDa → (Cut)
←75 kDa (Cut)
atg7 (78kDa)
75 kDa → (Cut)
←75 kDa (Cut)
atg12 (55kDa)
LC3-I (16kDa)
LC3-II(14kDa)
100 kDa → (Cut)
←100 kDa (Cut)
GRP78 (78kDa)
p-PERK (135kDa)
100 kDa →
75 kDa →
← 100 kDa
← 75 kDa
← 75 kDa
ATF6 (75kDa)
100 kDa →
←100 kDa
← 75 kDa (Cut)
IRE-1 (110kDa)
p-IRE-1 (110kDa)
100 kDa →
←100 kDa
75 kDa → (Cut)
← 75 kDa (Cut)
beclin-1 (60kDa)
1-Step Ultra TMB-Blotting Solution
M: Molecular marker; C: Control
M
Control 2wks
HFC 2wks
Control 8wks
HFC 8wks
C
135 kDa –
95 kDa →
VPS34 (100kDa)
95 kDa →
71 kDa →
UVRAG (75kDa)
230 kDa →
135 kDa →
95 kDa →
71 kDa →
←230 kDa
←135 kDa
←95 kDa
←71 kDa
PERK (135kDa)
45 kDa →
22 kDa →
←45 kDa
←22 kDa
GAPDH (37kDa)
71 kDa →
